# Long-Term Atrioventricular Block Following Valve Surgery: Electrocardiographic and Surgical Predictors

**DOI:** 10.3390/jcm13020538

**Published:** 2024-01-17

**Authors:** Jacopo Farina, Mauro Biffi, Gianluca Folesani, Luca Di Marco, Sofia Martin, Corrado Zenesini, Carlo Savini, Matteo Ziacchi, Igor Diemberger, Cristian Martignani, Davide Pacini

**Affiliations:** 1 Cardiology Unit, Arcispedale Sant’Anna, Azienda Ospedaliero-Universitaria di Ferrara, 44124 Ferrara, Italy; 2 Cardiology Unit, Cardiac Thoracic and Vascular Department, IRCCS Azienda Ospedaliero-Universitaria di Bologna, 40138 Bologna, Italy; mauro.biffi@aosp.bo.it (M.B.); matteo.ziacchi@aosp.bo.it (M.Z.); igor.diemberger@aosp.bo.it (I.D.); cristian.martignani@aosp.bo.it (C.M.); 3 Cardiac Surgery Unit, Cardiac Thoracic and Vascular Department, IRCCS Azienda Ospedaliero-Universitaria di Bologna, 40138 Bologna, Italy; gianluca.folesani@aosp.bo.it (G.F.); luca.dimarco@aosp.bo.it (L.D.M.); sofia.martin@aosp.bo.it (S.M.); carlo.savini@aosp.bo.it (C.S.); davide.pacini@aosp.bo.it (D.P.); 4 Epidemiology and Statistic Unit, IRCCS Istituto delle Scienze Neurologiche di Bologna, 40139 Bologna, Italy; corrado.zenesini@isnb.it

**Keywords:** cardiac valve surgery, pacemaker implantation, long-term follow-up

## Abstract

Background: Bradyarrhythmia requiring pacemaker implantation among patients undergoing valve surgery may occur even after several years, with unclear predictors. Our aim was to investigate the incidence of pacemaker implantation at different follow-up times and identify associated predictors. Methods: We conducted a retrospective study evaluating 1046 consecutive patients who underwent valve surgery at the Cardiac Surgery Division of Bologna University Hospital from 2005 to 2010. Results: During 10 ± 4 years of follow-up, 11.4% of these patients required pacemaker implantation. Interventions on both atrioventricular valves independently predicted long-term pacemaker implantation (SHR 2.1, 95% CI 1.2–3.8, *p* = 0.014). Preoperative atrioventricular conduction disease strongly predicted long-term atrioventricular block, with right bundle branch block as the major predictor (SHR 7.0, 95% CI 3.9–12.4, *p* < 0.001), followed by left bundle branch block (SHR 4.9, 95% CI 2.4–10.1, *p* < 0.001), and left anterior fascicular block (SHR 3.9, 95% CI 1.8–8.3, *p* < 0.001). Conclusion: Patients undergoing valvular surgery have a continuing risk of atrioventricular block late after surgery until the 12-month follow-up, which was clearly superior to the rate of atrioventricular block observed at long-term. Pre-operative atrioventricular conduction disease and combined surgery on both atrioventricular valves are strong predictors of atrioventricular block requiring pacemaker implantation.

## 1. Introduction

In patients undergoing cardiac valve surgery, atrioventricular block (AVB) occurs approximately in 20% of cases during the post-operative period [1,2,3,4]. Although most post-operative bradyarrhythmic episodes are transitory [5], a percentage ranging between 1.27% to 25.2% needs definitive pacemaker (PM) implantation [1,2,3,4,5,6,7].

This wide variability in incidence is due to the heterogeneity of the studies available in the literature. The majority analyzed the incidence of PM implantation exclusively in the postoperative period, while only two investigated the cumulative incidence of PM implantation during long-term follow-up [6,7]. These latter studies reported the highest PM implantation incidence but have some limitations: there was no evaluation of pre-operative electrocardiographic data, and considerable variability in terms of comorbidities and age was observed [6,7]. Moreover, there has been no focus on type of surgery, namely single-valve or multi-valve surgery [8,9,10,11,12,13,14,15,16,17].

As a result of these observations, it is possible to assume that AVB requiring PM therapy may occur even after several years, but it is unclear which type of valvular surgery is related to the highest risk of PM implantation [8,9,10,11,13,14].

Therefore, this study aims to analyze the incidence of PM implantation both on short- and long-term follow-up in patients undergoing valve surgery and to identify predictors of AVB requiring PM implantation.

## 2. Methods

The Strengthening the Reporting of Observational Studies in Epidemiology (STROBE) guidelines [18] were followed.

### 2.1. Study Design

This is a retrospective study of 1046 consecutive patients undergoing valve surgery, associated or not with other surgical procedures, from 2005 to 2010, at the Cardiac Surgery Unit of the IRCCS Azienda Ospedaliero-Universitaria di Bologna.

**Figure 1:**
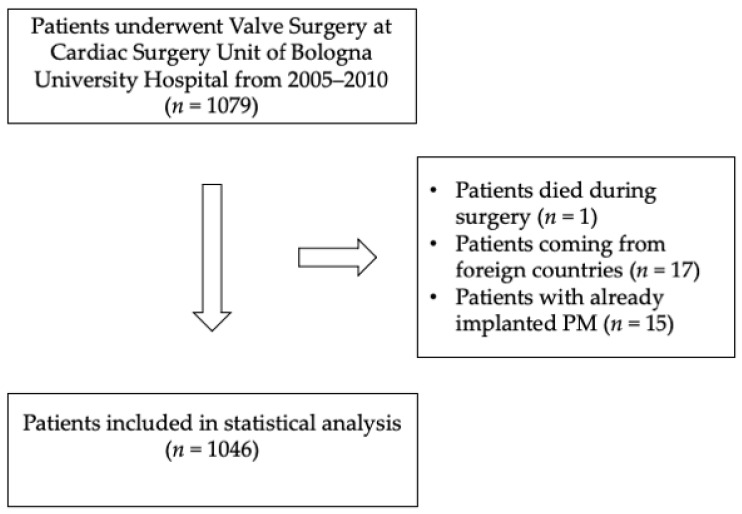
Flow chart of patients’ selection.

Patients with already implanted PMs, patients coming from foreign countries without the possibility of scheduling a follow-up, and those who died during surgery were excluded (Figure 1).

The study was conducted following the ethical principles of the Declaration of Helsinki. All patients were informed that their participation was voluntary, and all of them gave written informed consent. The ethical review boards of the participating hospital approved the study (registry FOR, 11/2009/U/Oss).

The primary endpoint was to evaluate the incidence of AVB requiring PM implantation in patients undergoing valve surgery, at different follow-up times (1 month, 1 year, 5 years, 10 years), and the secondary endpoint was to highlight predictors of PM implantation. As it is our customary approach to observe patients with postoperative AVB for recovery of intrinsic conduction at least 7 days after surgery, pacemaker recipients in this study had persistent AVB 2nd-3rd for a minimum of 8 days.

The study population was divided into five groups: (i) patients undergoing aortic valve surgery; (ii) patients undergoing mitral valve surgery; (iii) patients undergoing combined surgery for aortic and mitral valves; (iv) patients undergoing mitral and tricuspid valve surgery; (v) patients undergoing aortic, mitral and tricuspid valve surgery.

Data on cardiovascular risk factors, renal function and COPD prevalence were collected.

The pre-operative electrocardiographic data were analyzed by three independent

cardiologists: rhythm, duration of PR and QRS intervals, and morphology of ventricular depolarization waves were reported, referring to the last available electrocardiogram before surgery.

Pre-operative echocardiographic variables were also collected: grading of valvular disease severity, ventricular ejection fraction, end-diastolic and end-systolic volume were included in our analysis.

The surgical variables as type of prosthesis used, associated surgical procedures, and type of intervention performed, were recorded.

### 2.2. Clinical Follow-Up

Follow-up data were obtained via in-clinic visits, consultation of telematics health records and direct telephone contact for patients unable to attend ambulatory visits. In the event a PM, an ICD, or a CRT device had been implanted, the clinical indication was obtained. Only AVB requiring PM implantation was considered for the study endpoint. Sinus node disease and CRT indication without AVB were not considered.

### 2.3. Statistical Analysis

Continuous variables were tested for normal distribution with the Kolmogorov–Smirnov test. Non-normally distributed data were described as median value with interquartile range (IQR) and the Mann–Whitney U test was used. Categorical variables were summarized in terms of counts (*n*) and percentages (%) and were compared using chi-squared test and the p-value was adjusted with Bonferroni correction in the case of multiple comparisons.

Competing-risks regression based on Fine and Gray’s proportional sub-hazard model were performed to identify predictors of PM implantation. The time to enter in the analysis was the date of surgery and the time to endpoint was the date of PM implantation or the date of the last follow-up information, whichever came first. Death was the competitive risk of the PM implantation. Variables showing a *p*-value of < 0.05 were included in the multivariable model. Log-likelihood ratio test was used to select the best multivariable model. The results were presented with Sub-Hazard Ratio (SHR) and respective 95% confidence intervals (95% CI). All tests were 2-sided, and the statistical significance was defined as *p* < 0.05.

The statistical analyses were performed with Stata SE 14.2 software.

## 3. Results

### 3.1. Clinical Characteristics

Overall, 1046 patients were considered (61.8% male, median age 63 years). Clinical, electrocardiographic, echocardiographic, and operating characteristics are shown in Table 1.

Patients receiving PMs (PM+ group) were significantly older than those not receiving PMs (PM− group).

Sex and cardiovascular risk factors did not differ among the two groups. However, COPD was significantly more prevalent in the PM+ group, and patients with implanted PMs had lower eGFR (Table 1).

### 3.2. Pre-Operative Electrocardiographic Characteristics

The overall prevalence of atrial fibrillation (AF)/Flutter rhythm was 24.2% and it was significantly higher in the PM+ group than the PM− group (34.1% vs. 23.3% respectively, *p* = 0.022).

The PR interval and the QRS duration was longer in PM+ patients (Table 1). Also, first-degree AVB and bundle-branch blocks (BBB) were significantly more prevalent in PM+ patients (26.7% vs. 9.6% for first degree AVB, *p* < 0.001; 45.1% vs. 16.9% for BBB, *p* < 0.001).

The most frequent intraventricular delays in the PM+ group were: RBBB; (22.0% vs. 4.3%, *p* < 0.001), LBBB; (11% vs. 3.6%, *p* < 0.001), LAFB (8.8% vs. 3.6%, *p* = 0.121), bifascicular block (RBBB + LAFB; 5.5% vs. 0.8%, *p* = 0.001), non-specific delay (5.5% vs. 2.8%, *p* = 0.158) and incomplete LBBB (1.1% vs. 0.4%, *p* = 0.369).

### 3.3. Surgical Characteristics

Most patients (72.6%) received single valvular surgery, about a quarter (22.2%) underwent bi-valvular surgery, and 5.1% underwent triple valve surgery (Table 2).

Regarding the used technique for mitral intervention, valve replacement occurred in 99% while mitral valve repair occurred in 1%.

For tricuspid intervention, all patients were treated with valve repair, in 29.7% annuloplasty occurred, in 70.3% the repair was done without the annuloplasty technique.

**Figure 2:**
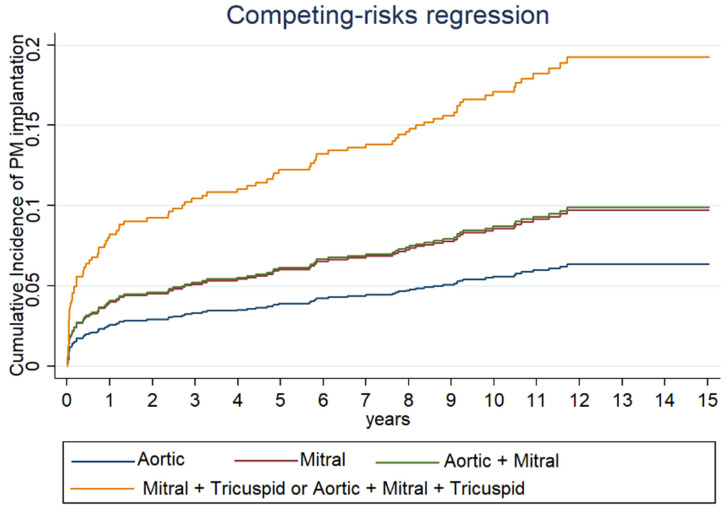
Cumulative incidence of PM implantation at different time of follow-up for each type of valve surgery estimated with Fine and Gray method.

PM implantation was significantly more frequent in patients undergoing multivalvular procedures compared to single-valve surgery independently of the type of single-valve surgery and of the prosthetic material (biological vs. mechanical valve) (Table 2, Figure 2).

### 3.4. Incidence of PM Implantation

Of the 1046 patients at baseline, 735 (70%) reached a 10-year follow-up, and 11.4% required PM implantation along 10 ± 4 years of follow-up. 

The rate of PM implantation was 1.8% at hospital discharge, 4% at the first year of follow-up, 5.6% at the fifth year, and 11.4% at ten years (Table 3).

Regarding the indication for PM implantation, all patients included in the analysis received PMs due to AVB. Patients who were implanted for other indications were excluded. Specifically, ninety-one patients (11.4%) received PMs due to AVB. Among them, three received a cardiac resynchronization therapy (CRT) pacemaker due to EF < 35% with complete AVB.

Additionally, one patient received a CRT defibrillator as a Class 1 indication but without AVB, and another patient was implanted due to sinus node disease. These two patients were excluded from the endpoint analysis.

### 3.5. Predictors of PM Implantation at Univariate Analysis

At univariate analysis, age was a predictor of PM implantation. Subjects between 64-72 years old and subjects over 72 years old have a considerably higher risk of PM implantation than younger patients (Table 4). PM implantation increased continuously with advancing age.

Electrocardiographic measures of atrioventricular conduction time were the most powerful pre-operative predictors of PM implantation (Table 4). A 1st degree AV block and an AF/Flutter rhythm were also predictive of PM implantation at follow-up (Table 4).

Regarding echocardiographic values, there was no difference between the two groups in terms of ejection fraction, end-diastolic volume and end-systolic volume (Table 4).

Considering the number of treated valves, surgery on three valves or two valves was a predictor of PM implantation compared to single-valve interventions (Table 4). When considering the types of valves treated, surgery including both atrioventricular valves carried a higher risk of PM implantation (Table 4). 

Intervention involving the mitral valve, if not associated with the treatment of the tricuspid valve, was not predictive for PM implantation (Table 4).

### 3.6. Predictors of PM Implantation at Multivariate Analysis

The multivariate analysis confirmed electrocardiographic measures of atrioventricular conduction disease as independent predictors of PM implantation (Table 5), with RBBB as the major predictor (SHR 7.0, 95% CI 3.9–12.4, *p* < 0.001).

Age as a continuous variable was also predictive of PM implantation (Table 5).

Surgery involving both atrioventricular valves was the most powerful predictor among surgical variables (SHR 2.1, IC 95% 1.2–3.8, *p* = 0.014).

### 3.7. Survival

Patients undergoing definitive PM implantation had lower but not statistically significant survival (13.1 ± 0.1 years vs. 12.8 ± 0.4 years respectively in PM− group and PM+ group).

## 4. Discussion

The rate of PM implantation after valve surgery at 1 month, 1 year, 5 years, and 10 years were analyzed to observe the occurrence of AVB over time. The most powerful predictors of AVB observed in our study were pre-existing atrioventricular conduction disorders, and surgery on both atrioventricular valves.

The incidence of AVB requiring permanent PMs in the first month and the first year of follow-up was, respectively, 1.8% and 4%, while the incidence at the fifth year of follow-up was 5.6%. These data confirm the hypothesis that the first year after valve surgery represents a high-risk time frame for progression/onset of atrioventricular conduction disease.

The risk of such an unwanted event is more commonly the progression of a pre-existent conduction disease rather than a new-onset AVB related to surgery and is more likely to occur in patients undergoing extensive valve surgery on the atrioventricular valves (Table 5), on whose anatomic skeleton the conduction system is embedded. Secondly, its progression increases with time, reaching 11.4% in the ten-year follow-up (Table 3).

Patients undergoing valve surgery have a greater propensity to develop high grade AVB requiring pacemaker therapy compared to the general population [19,20]. 

**Figure 3:**
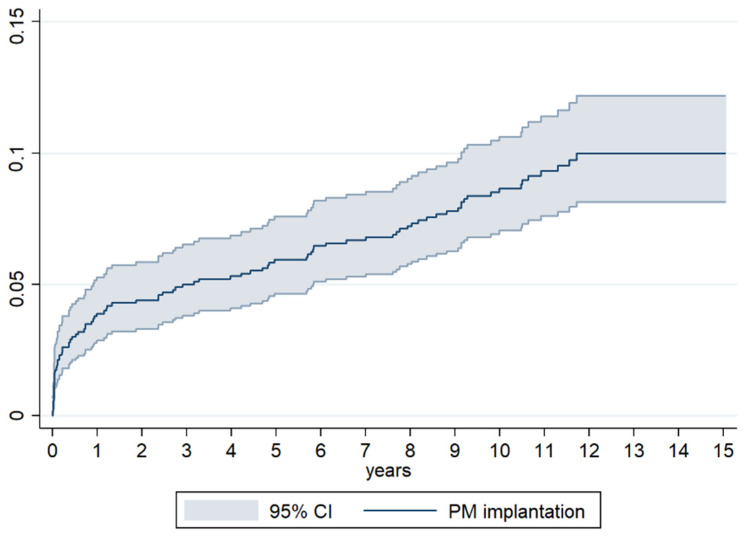
Cumulative incidence of Pacemaker implantation at different time of follow-up after surgery.

Valve surgery can be associated with an injury of the cardiac conduction system, an occurrence that may not be limited to the immediate peri-operative period but extend also to the first year after surgery, as per our data (Figure 3). Indeed, the implantation rate in our patients had a different slope in the first year compared to the long-term follow-up: while it increased more than two-fold in the 11 months after hospital discharge, it only doubled in the following 9 years.

Preoperative functional characteristics of the cardiac conduction system represent the major risk factor for PM implantation in the long term, this latter increasing with more extensive fascicular blocks (Table 5).

RBBB (SHR 7, IC 95% 3.9–12.4, *p* < 0.001) and bifascicular block (SHR 7.1, IC 95% 2.8–19.8, *p* < 0.001) were its most significant independent predictive factors. About a third of patients with a pre-operative RBBB subsequently received PM implantation, while a higher rate is reported for patients with pre-operative bifascicular block, an occurrence in line with Koplan et al.’s [15] observation.

This is consistent with the notion that any injury to the left-sided posterior fascicle of the left branch is more likely to cause high-grade AVB in patients who already have RBBB, as learnt in previous observations and in transcatheter aortic valve replacement procedures [15,21,22].

First degree AVB may also be associated with a higher incidence of PM implantation [15], but though observed in the univariate analysis (Table 4), it was not found as an independent predictor in our study; it should probably be considered in the context of the specific valve surgery. 

In single-valve surgery, aortic surgery had the lowest risk of PM implantation at a 10-year follow-up (8.1%), while mitral valve surgery reached 11%, though not statistically significant (SHR 1.2, IC 95% 0.7–2.2, *p* = 0.52). This trend was also previously highlighted by Leyva et al., who observed a higher implantation rate than aortic valve surgery in a maximum 14-year follow-up [6].

Most of the studies reported higher PM implantation rates for aortic valve surgery but the number of studies conducted on mitral valve surgery is lower [1,4,5,7,8,9,11,13,14,15,17].

A Scandinavian study on patients undergoing isolated aortic valve surgery with a 10-year follow-up reported a PM implantation rate of 12% [7], far greater than ours. The difference may be related to the older age than our patients (71 vs. 60 years old on average), since age in itself is predictor of PM implantation (Table 5). Similar considerations can be applied to Leyva et al.’s study, where substantial differences are observed amongst subgroups in terms of age and comorbidities [6]. Older age in aortic surgery recipients is associated with a greater extent of conduction system disease, thus explaining the incidence of AVB requiring PM therapy compared to younger mitral surgery recipients, who on the contrary are at higher risk of AVB due to proximity to the conduction system location.

In our study, we observed that multivalvular interventions (especially triple and double valve surgeries including mitral and tricuspid valves) have considerably higher implantation rates at 10 years compared to those reported for other interventions (Figure 2). This suggests that surgery of both atrioventricular valves and number of treated valves considerably increase the risk of PM implantation, as reported by other data in the literature [2,11,12,13,15]. Indeed, combined mitro-aortic valve surgery had a significantly lower incidence of PM implantation at 10 years (12%), when compared to combined atrioventricular valve surgery (23.1%) and triple valve surgery (29.7%).

Therefore, the number and type of valves treated strongly influence the risk of AVB at follow-up, treated valves impacting more than valve number; because of the anatomical relationship with the conduction system, mitral-tricuspid surgery carries the highest predictive risk of PM implantation (SHR 2.1, IC 95% 1.2–3.8, *p* = 0.014).

Eventually, our observation confirms age as a predictor of PM implantation, as observed in other studies [6,9,13,15] and may explain differences in the incidence of PM implantation across valve surgery subgroups, aortic valve replacement recipients being older than mitral surgery recipients.

It stems from our data that a more extensive surgery on the heart skeleton, such as involving both atrioventricular valves, and an advanced pre-operative conduction disorder help to identify those patients more likely to develop AVB along the first-year follow-up. Indeed, patients with a similar extent of tricuspid or mitral valve disease but with a more severe clinical profile and a much older age than ours, treated via trans-catheter edge-to-edge valve repair, had no risk of developing AVB, as no direct action on the conduction system occurs in that setting [23,24,25]. 

We believe that our observations are helpful to risk-stratify valve surgery recipients for the risk of AVB requiring PM therapy in the long term, where coexistence of pre-operative RBBB or bifascicular block in the setting of combined mitral-tricuspid surgery or triple surgery may strongly suggest prophylactic epicardic electrode placement during surgery to manage AVB postoperatively. This strategy enables a minimal risk of PM-related complications owing to the absence of intravascular hardware, endovascular infections of implantable electronic devices being a life-threatening event, as known from the literature [22]. Moreover, the recent ESC guidelines emphasize the importance of avoiding trans-valvular lead placement in patients operated on the tricuspid valve or with tricuspid regurgitation to minimize dreadful events at follow-up via the use of epicardic or coronary sinus lead implantation [26,27,28].

## 5. Limitations

Most of the limitations of this study are due to its retrospective and observational nature. Compared to other studies, we collected more information regarding pre-operative electrocardiographic data, surgical variables, and comorbidities on all consecutive valve surgery recipients, which strengthens the power of our investigation, though the number of combined valve surgery interventions is limited.

## 6. Conclusions

The occurrence of AVB is not limited to the early postoperative phase but extends to the first year after surgery. Interventions involving both atrioventricular valves pose the highest risk of high-grade AVB both at 12 months and in the long term.

Preoperative atrioventricular conduction disorders are strongly predictive of long-term AVB: RBBB with or without LAFB is the major predictor, followed by LBBB and LAFB. This aspect is independent of age, which also increases the risk of AVB requiring PM, as in the general population. 

Therefore, when planning a combined mitral-tricuspid or triple-valve surgery in patients older than 70 years with high-risk atrioventricular conduction disorders, the risk of progression to complete AVB is anticipated.

## Figures and Tables

**Table 1:** Clinical, echocardiographic and electrocardiographic characteristics of patients at baseline (surgery).

Variables	Overall (*n* = 1046) ^1^	PM− (*n* = 955) ^1^	PM+ (*n* = 91) ^1^	*p*-Value ^2^
Clinical				
Age, years	63 (53–72)	62 (52–71)	69 (60–74)	<0.001
Sex male	646/1046 (61.8)	596/955 (62.4)	54/91 (55.0)	0.162
Hypertension	666/1046 (63.7)	606/955 (63.5)	60/91 (65.9)	0.639
Dyslipidaemia	346/1046 (33.1)	312/955 (32.7)	34/91 (37.4)	0.363
Diabetes	921/1045 (11.9)	108/954 (11.3)	16/91 (17.6)	0.078
Smoke	323/1046 (30.9)	288/955 (30.2)	35/91 (38.5)	0.101
COPD	82/1046 (7.8)	70/955 (7.3)	12/91 (13.2)	0.041
Creatinine, mg/dL	1.0 (0.9–1.2)	1.0 (0.9–1.2)	1.1 (0.9–1.4)	0.013
GFR, mL/min/1.73 m^2^	70 (53–89)	71 (54–90)	61 (44–80)	<0.001
Echocardiography				
EF, %	61 (55–65)	61 (55–66)	60 (55–65)	0.509
EDV, mL	121 (87–156)	120 (87–155)	141 (97–166)	0.161
ESV, mL	44 (29–70)	44 (29–67)	50 (32–74)	0.194
Electrocardiography				
AF/Flutter	253/1045 (24.2)	222/954 (23.3)	31/91 (34.1)	0.022
PR duration, ms	170 (160–180)	170 (160–180)	180 (168–205)	<0.001
QRS duration, ms	100 (90–110)	100 (90–110)	110 (90–130)	<0.001
PR > 200 ms	86/793 (10.8)	70/733 (9.6)	16/60 (26.7)	<0.001
QRS morphology				
Normal	835/1046 (79.8)	794/955 (83.1)	41/91 (45.1)	<0.001
RBBB	61/1046 (5.8)	41/955 (4.3)	20/91 (22.0)	<0.001
LBBB	44/1046 (4.2)	34/955 (3.6)	10/91 (11.0)	0.006
LAFB	42/1046 (4.0)	34/955 (3.6)	8/91 (8.8)	0.121
RBBB and LAFB	13/1046 (1.2)	8/955 (0.8)	5/91 (5.5)	0.001
Non-specific delay	32/1046 (3.1)	27/955 (2.8)	5/91 (5.5)	0.158
Incomplete RBBB	14/1046 (1.3)	13/955 (1.4)	1/91 (1.1)	0.835
Incomplete LBBB	4/1046 (0.5)	4/955 (0.4)	1/91 (1.1)	0.369

AF: atrial fibrillation; COPD: chronic obstructive pulmonary disease; EDV: end diastolic volume; EF: ejection fraction; SV: end systolic volume; GFR: glomerular filtration rate; LAFB: left anterior fascicular block; LBBB: left bundle branch block; RBBB: right bundle branch block. ^1^ median (interquartile range) for continuous variables and number (%) for categorical variables. ^2^ Mann–Whitney U test for continuous variables and chi-square test for categorical variables.

**Table 2:** Surgical characteristics.

Variables	Overall (*n* = 1046) ^1^	PM− (*n* = 955) ^1^	PM+ (*n* = 91) ^1^	*p*-Value ^2^
Indication to surgery				0.637
Valve disease	822/1046 (78.6)	746/955 (78.1)	76/91 (83.5)	
AA + Valve disease	154/1046 (14.7)	144/955 (15.1)	10/91 (11.0)	
Endocarditis	34/1046 (3.2)	31/955 (3.2)	3/91 (3.3)	
Aortic Dissection	36/1046 (3.4)	34/955 (3.5)	2/91 (2.2)	
Number of valves treated				
1	759/1046 (72.6)	707/955 (74.0)	52/91 (57.1)	0.002
2	234/1046 (22.4)	206/955 (21.6)	28/91 (30.8)	0.133
3	53/1046 (5.1)	42/955 (4.4)	11/91 (12.1)	0.001
Valves treated				
Aortic ^3^	536/1046 (51.2)	504/955 (52.8)	32/91 (35.2)	0.007
Mitral	228/1046 (21.8)	207/955 (21.7)	21/91 (23.1)	0.757
Aortic and Mitral	151/1046 (14.4)	137/955 (14.4)	14/91 (15.4)	0.788
Mitral and Tricuspid	77/1046 (7.4)	64/955 (6.7)	13/91 (14.3)	0.041
Aortic, Mitral, and Tricuspid	54/1046 (5.2)	43/955 (4.5)	11/91 (12.1)	0.009
Prosthesis				0.052
Mechanical	726/1046 (69.4)	671/955 (70.3)	55/91(60.4)	
Biological	320/1046 (30.6)	284/955 (29.7)	37/91 (39.6)	
CABG	126/1046 (12.1)	111/955 (11.7)	15/91 (15.4)	0.306
Maze	125/1046 (12.0)	110/955 (11.5)	15/91 (16.5)	0.163
Other	322/1046 (30.8)	303/955 (31.7)	19/91 (20.9)	0.032
Type of Mitral valve intervention ^4^				0.552
Mitral replacement	506/511 (99.0)	448/452 (99.1)	58/59 (98.3)	
Mitral repair	5/511 (1.0)	4/452 (0.9)	1/59 (1.7)	
Type of Tricuspid valve intervention ^5^				0.498
Tricuspid repair	92/131 (70.3)	76/106 (71.6)	16/25 (64.0)	
Tricuspid annuloplasty	39/131 (29.7)	30/106 (28.4)	9/25 (36.0)	

AA: Aortic aneurism; CABG: coronary artery bypass graft. ^1^ Number (%) for categorical variables. ^2^ Mann–Whitney U test for continuous variables and chi-square test for categorical variables. ^3^ Every aortic valve intervention consisted of aortic valve replacement. ^4^ Overall number refers to patients that received intervention on Mitral valves. ^5^ Overall number refers to patients that received intervention on Tricuspid valve.

**Table 3:** Cumulative incidence of Pacemaker implantation at different time of follow-up after surgery.

Valve Surgery	PM Implantation at 1 Month	PM Implantation at 1 Year	PM Implantation at 5 Years	PM Implantation at 10 Years
Aortic	4/530 = 0.8%	12/523 = 2.3%	20/499 = 4.0%	31/382 = 8.1%
Mitral	7/224 = 3.1%	10/214 = 4.7%	15/209 = 7.2%	18/164 = 11.0%
Aortic + Mitral	4/147 = 2.7%	7/146 = 4.8%	10/134 = 7.5%	12/100 = 12.0%
Mitral + Tricuspid	1/75 = 1.3%	6/71 = 8.5%	8/65 = 12.3%	12/52 = 23.1%
Aortic + Mitral + Tricuspid	3/54 = 5.6%	5/53 = 9.4%	7/52 = 13.5%	11/37 = 29.7%
Overall	19/1030 = 1.8%	40/1007 = 4.0%	60/959 = 5.6%	84/735 = 11.4%

**Table 4:** Univariable Cox regression models using time to implantation as dependent variable.

Variables	Sub-Hazard Ratio	95% CI	*p*-Value
Age-continuous one-year increase Age-category	1.03	1.01–1.05	<0.001
<54 years	reference	-	-
54–63 years	1.5	0.7–3.2	0.328
64–72 years	3.3	1.7–6.5	0.001
>72 year	3.1	1.6–6.3	0.001
QRS morphology			
Normal	reference	-	-
RBBB	7.8	4.6–13.3	<0.001
LBBB	5.1	2.6–10.3	<0.001
LAFB	4.3	2.0–9.0	<0.001
RBBB and LAFB	10.1	3.9–26.2	<0.001
Non-specific delay	3.4	1.4–8.5	0.009
Incomplete RBBB	1.5	0.2–11.4	0.671
Incomplete LBBB	4.0	0.6–25.1	0.136
PR > 200 ms	3.2	1.8–5.7	<0.001
AF/Flutter	1.8	1.1–2.7	0.011
COPD	1.9	1.01–3.4	0.047
Echocardiography			
EF, %	1.0	0.97–1.01	0.599
EDV, mL	1.0	0.99–1.01	0.265
EDS, mL	1.0	0.99–1.01	0.316
Number of valve treated			
1	reference	-	-
2	1.8	1.1–2.9	0.012
3	3.3	1.7–6.3	<0.001
Type of valve surgery			
Aortic	reference	-	-
Mitral	1.6	0.9–2.7	0.114
Aortic+Mitral	1.6	0.8–3.0	0.150
Aortic + Mitral + Tricuspid or Mitral + Tricuspid	3.3	1.9–5.5	<0.001
Interventions involving mitral valve vs. other interventions	2.0	1.3–3.1	0.001
Interventions involving tricuspid valve vs. other interventions	2.6	1.7–4.2	<0.001

AF: atrial fibrillation; COPD: chronic obstructive pulmonary disease; EDV: end diastolic volume; EF: ejection fraction; ESV: end systolic volume; LAFB: left anterior fascicular block; LBBB: left bundle branch block; RBBB: right bundle branch block.

**Table 5:** Multivariable estimated with Fine and Gray model using time to implantation as dependent variable.

Variables	Sub-Hazard Ratio	95% Confidence Interval	*p*-Value
Age-continuous one-year increase	1.02	1.01–1.03	0.045
QRS morphology			
Normal	reference	-	-
RBBB	7.0	3.9–12.4	<0.001
LBBB	4.9	2.4–10.1	<0.001
LAFB	3.9	1.8–8.3	0.001
RBBB and LAFB	7.1	2.5–19.8	<0.001
Non-specific delay	3.4	1.3–8.8	0.010
Incomplete RBBB	1.2	0.2–8.5	0.890
Incomplete LBBB	2.7	0.5–13.8	0.245
Type of valve surgery			
Aortic	reference	-	-
Mitral	1.2	0.7–2.2	0.522
Aortic + Mitral	1.5	0.8–2.8	0.217
Aortic + Mitral + Tricuspid or Mitral + Tricuspid	2.1	1.2–3.8	0.014

LAFB: left anterior fascicular block; LBBB: left bundle branch block; RBBB: right bundle branch block.

## Data Availability

The datasets generated during and/or analyzed during the current study are available from the corresponding author upon reasonable request. The data are not publicly available due to privacy regulations.
